# Sub-lethal ionizing radiation alters Foxp3 expression in CD4+ T regulatory (Treg) cells in vitro and in vivo

**DOI:** 10.1186/2051-1426-3-S2-P269

**Published:** 2015-11-04

**Authors:** Anita Kumari, Samantha Simon, Charlie Garnett-Benson

**Affiliations:** 1 GSU, Atlanta, GA, USA

## 

The use of sub-lethal radiation has been shown to alter cell phenotype and gene expression. T regulatory (Treg) cells are phenotypically defined as being CD4+CD25+ Foxp3+ T cells and are known to suppress the function of CD8+ cytotoxic T lymphocytes (CTLs). Inhibiting the suppressive function of Treg cells allows for activation and proliferation of CD8+ CTLs. We examined the effects of sub-lethal radiation on Tregs 24- to 72-hrs post-treatment. We found that radiation treatment decreased the number of Treg cells however the total CD4+ T cell fraction remained unaltered. Our data suggests that the use of sub-lethal radiation can modulate the expression of Foxp3 in CD4+ Treg cells *in vitro* and *in vivo*. Moreover, the loss of Foxp3-mediated suppressive functions may be linked to increased CTL activity.

